# Storage-Induced Platelet Apoptosis Is a Potential Risk Factor for Alloimmunization Upon Platelet Transfusion

**DOI:** 10.3389/fimmu.2018.01251

**Published:** 2018-06-05

**Authors:** Anno Saris, Ivan Peyron, Pieter F. van der Meer, Tor B. Stuge, Jaap Jan Zwaginga, S. Marieke van Ham, Anja ten Brinke

**Affiliations:** ^1^Department of Immunopathology, Sanquin Research, Amsterdam, Netherlands; ^2^Landsteiner Laboratory, Academic Medical Centre, University of Amsterdam, Amsterdam, Netherlands; ^3^Department of Plasma Proteins, Sanquin Research, Amsterdam, Netherlands; ^4^Product and Process Development, Sanquin Blood Bank, Amsterdam, Netherlands; ^5^Immunology Research Group, Department of Medical Biology, University of Tromsø – The Arctic University of Norway, Tromso, Norway; ^6^Department of Immunohematology and Blood Transfusion, Leiden University Medical Center, Leiden, Netherlands; ^7^Swammerdam Institute for Life Sciences, University of Amsterdam, Amsterdam, Netherlands

**Keywords:** platelet, transfusion medicine, alloimmunization, storage, internalization, antigen presentation

## Abstract

Platelet transfusion can elicit alloimmune responses leading to alloantibody formation against donor-specific polymorphic residues, ultimately resulting in platelet transfusion refractoriness. Universal leukoreduction significantly reduced the frequency of alloimmunization after platelet transfusion, thereby showing the importance of white blood cells (WBCs) in inducing this alloresponse. It is, however, unknown if the residual risk for alloimmunization is caused by WBCs remaining after leukoreduction or if alloimmunization can be induced by platelets themselves. This study investigated the capacity of platelets to induce alloimmunization and identified potential product-related risk factors for alloimmunization. First, internalization of allogeneic platelets by dendritic cells (DCs) was demonstrated by confocal microscopy. Second, after internalization, presentation of platelet-derived peptides was shown by mass spectrometry analysis of human leukocytes antigen (HLA)-DR eluted peptides. Third, platelet-loaded DCs induced platelet-specific CD4 T cell responses. Altogether, this indicates a platelet-specific ability to induce alloimmunization. Therefore, factors enhancing platelet internalization may be identified as risk factor for alloimmunization by platelet concentrates. To investigate if storage of platelets is such a risk factor, internalization of stored platelets was compared with fresh platelets and showed enhanced internalization of stored platelets. Storage-induced apoptosis and accompanied phosphatidylserine exposure seemed to be instrumental for this. Indeed, DCs pre-incubated with apoptotic platelets induced the strongest IFN-γ production by CD4 T cells compared with pre-incubation with untreated or activated platelets. In conclusion, this study shows the capacity of platelets to induce platelet-specific alloimmune responses. Furthermore, storage-induced apoptosis of platelets is identified as potential risk factor for alloimmunization after platelet transfusions.

## Introduction

The effectiveness of prophylactic platelet transfusions can be monitored by determining the increase in circulating platelets after transfusion (the count increments), prevention of bleeding and/or the time to the next transfusion. If patients display multiple subsequent ineffective transfusions they are considered to be refractory for platelet transfusion. This is a frequent clinical problem, which is observed in 7–37% of hemato-oncological patients receiving multiple platelet transfusions (1–3). Platelet refractoriness is associated with prolonged hospital stay, increased risk for bleeding and reduced survival (3, 4). Next to non-immune factors, immune-mediated refractoriness to platelet transfusions occurs in 5–15% of all patients receiving platelets transfusions (2, 5, 6). This is most frequently caused by alloimmunization of the recipient against non-matching human platelet antigens (HPA) or human leukocytes antigens (HLA) class I that are recognized as foreign by the recipient (6, 7). The resulting alloantibodies can induce platelet transfusion refractoriness, especially when targeting HLA class I antigens (6). Besides causing refractoriness, HLA class I alloimmunization complicates curative stem cell therapies (2).

Although alloimmunization after platelet transfusions is suggested to be initiated *via* either the direct or indirect pathway of allorecognition (6), the indirect pathway seems to be dominant for humoral alloimmunization (1, 8). IgG alloimmunization *via* the indirect pathway is initiated when antigen-presenting cells are stimulated by nitric oxide to process and present allogeneic antigens (8–10). These presented allopeptide–MHC complexes must be recognized by CD4 T cells (11), to subsequently provide help to alloreactive B cells as necessary step in the eventual production of alloantibodies. Directed against transfused platelets, such alloantibodies result in rapid clearance of antibody opsonized platelets, rendering subsequent transfusions more and more ineffective (12, 13). It is known that previous sensitization (e.g., by pregnancy) and repeated exposures to platelet concentrates increase the risk for alloimmunization (2, 6), but knowledge regarding patient and product-related risk factors for alloimmunization remains very limited.

Most interesting in this respect is that even though platelets are most abundant, the contaminating white blood cells (WBCs) in platelet concentrates (PCs) are so far held mainly responsible for the induction of alloimmunization (2, 6, 14–16). Indeed, in early days, alloimmunization after platelet transfusion seemed completely prevented when PCs were depleted from contaminating WBCs (17–19). These findings led to the proposal that platelets themselves are incapable of inducing alloimmunization (17, 18, 20). By contrast, more recent animal studies suggest that platelets themselves are capable of inducing alloimmune responses against HLA class I (8–10); however, residual WBCs were still present in platelet products making it impossible to dissect the role of platelets and WBCs in the observed alloimmune responses. Although universal leukoreduction significantly reduced alloimmunization after platelet transfusions *in vivo* (1, 2), alloimmunization still occurs and it is unknown whether this is caused by WBCs still contaminating platelet concentrates or also by platelets themselves.

Alloantibodies induced by platelet transfusions are most frequently directed against HLA class I (15, 21), but platelets additionally express various molecules with polymorphisms, known as HPAs, against which alloantibodies can also be formed. Currently, 33 HPAs are described which are expressed on 6 different glycoproteins (22, 23). Expression of HPAs, however, is not limited to platelets, as for example HPA-5 and -13 are in glycoprotein Ia/CD49b, which can also be expressed by T, B, and NK cells (24, 25). Furthermore, the expression of glycoprotein IIIa/CD61, which contains seven HPAs, is also reported for endothelial cells, trophoblasts, and osteoclasts (23, 26–28). Therefore, although immunization against HPA suggests that platelets themselves can induce alloimmunization, this may be mediated by other cells.

This study investigates the capacity of platelets to induce alloimmune responses and identifies potential product-related risk factors for alloimmunization after platelet transfusion. In this respect, we show internalization of allogeneic platelets by dendritic cells (DCs), which is significantly enhanced after storage. This internalization results in presentation of platelet-derived peptides by DCs and subsequent platelet-specific CD4 T cell responses.

## Materials and Methods

### Human Blood Samples

Leukapheresis products or whole blood derived buffy coats obtained from anonymized Sanquin blood donors after giving written informed consent were used to isolate monocytes from random or HLA-DRB3*01:01-positive individuals. Citrated whole blood was obtained to isolate HPA-1a^+^, HPA-1a^−^, or fresh platelets. Full blood donations from anonymized Sanquin blood donors were used to prepare platelet concentrates. All was approved by the Sanquin Ethical Advisory Board and in accordance with the declaration of Helsinki and according to Dutch regulations.

### Platelet Concentrates

Buffy coats were obtained from full blood donations as previously described (29). Platelet concentrates (PCs) were prepared from five pooled buffy coats, and 1 U of plasma obtained from one of these donors. After pooling and 4.5 min centrifugation at 1,940 *g*, platelet-rich plasma was transferred *via* a leukoreduction filter to a storage container using an automated separator (Compomat, Fresenius, Emmer Compascuum, The Netherlands). Products were left either untreated (control) or were treated with Mirasol pathogen reduction technology (TerumoBCT, Zaventem, Belgium). Mirasol-treated products were always produced in parallel with a paired untreated unit, and therefore two ABO-identical platelet concentrates were pooled, mixed, and subsequently split to allow a paired comparison. Mirasol treatment was performed according to the manufacturer’s instructions. In short, 50 mM riboflavin was added to PCs, which were subsequently UV-B illuminated for 5–10 min with 6.2 J/ml. All products were stored at room temperature with gentle agitation, up to a maximum of 15 days [7 days is the current standard in the Netherlands, but we also wanted to investigate more exceptional cases and transfused platelets remain in circulation up to 15 days after collection (30)]. All PCs were tested negative for bacterial contamination using BactAlert (bioMérieux, Marcy-l’Étoile, France) and fulfilled standard quality requirements (i.e., <1 × 10^6^ leukocytes/product, visible swirl “not red,” >250 × 10^9^ platelet/product, 150–400 ml total volume).

### Platelet Preparations and Membrane Labeling

Platelet rich plasma was obtained by centrifugation of freshly drawn citrated blood from healthy individuals at 200 *g* for 10 min or by taking 10 ml samples from PCs on day 1, day 7, and/or day 15 of storage. Subsequently, platelets were washed, and if indicated, labeled 20 min in PBS using 3.75 µM PKH26 cell membrane dye (Sigma Aldrich, Zwijndrecht, The Netherlands) under continuous gentle agitation, after which platelets were washed once using sequestrine buffer [17.5 mM Na_2_HPO_4_, 8.9 mM Na_2_EDTA, 154 mM NaCl, pH 6.9, containing 0.1% (wt/vol) bovine serum albumin, all obtained from Merck Millipore, Amsterdam, The Netherlands] supplemented with 10% heat inactivated fetal calf serum (Bodinco, Alkmaar, The Netherlands). If indicated, platelets were left untreated, activated using 100 µM thrombin receptor activator peptide 6 amide (Bachem, Bubendorf, Switzerland), made apoptotic using 5 µM Calimycin A23187 (Sigma Aldrich) as previously described (31, 32). Alternatively, platelets were incubated with either 250 µg/ml purified bovine phosphatidylserine (PS) or phosphatidylcholine (both from Sigma Aldrich), which was shown to result in incorporation in the outer membrane of RBCs (33). After 20 min, platelets were washed using sequestrine buffer, centrifuged at 1,600 *g* for 5 min and dissolved in Cellgro DC serum-free medium (Cellgenix, Freiburg, Germany). To determine the effects of pathogen reduction, activation or apoptosis induction and exposure to purified PS or phosphatidylcholine, 0.5 × 10^6^ platelets were stained either in PBS with phycoerythrin-cyanine 7-labeled anti-CD61 (Beckman Coulter, Woerden, The Netherlands) combined with either fluorescein isothiocyanate (FITC)-labeled anti-CD62P (Beckman Coulter) or phycoerythrin-cyanine 5-labeled anti-HLA class I (clone W6-32, BioLegend, London, UK) or in annexin V binding buffer (BioLegend) with allophycocyanin (APC)-labeled Annexin V (BD Biosciences, Breda, The Netherlands).

### Human DCs

Monocytes were isolated either from fresh leukapheresis material using the Elutra Cell Separation System (Gambro, Lakewood, CO, USA) or from HLA-typed buffy coats using Ficoll density centrifugation and CD14 microbead MACS isolation (Miltenyi, Leiden, The Netherlands) as previously described (34, 35), and subsequently frozen until further use. Purity of monocytes was determined using flow cytometry with APC-labeled anti-CD14, PE-labeled anti-CD3, and FITC-labeled anti-CD66b (all from BD Biosciences) and was >90%. On day 0, monocytes were thawed and differentiated into DCs by culturing 1 × 10^6^ monocytes/ml in a T75 culture flask or 6-well plate (both from Nunc/Sanbio BV, Uden, The Netherlands) in 20 or 2 ml of Cellgro DC serum-free medium (Cellgenix), respectively, supplemented with 800 IU/ml IL4 and 1,000 IU/ml granulocyte–macrophage CSF (both from Cellgenix, Freiburg, Germany), 100 U/ml penicillin, and 100 U/ml streptomycin (both from Gibco/Thermo Fischer Scientific, The Netherlands) at 37°C and 5% CO_2_ for 6–7 days.

### Platelet Internalization by DCs

DCs were harvested and incubated 0.5–4 h in 1:5–1:80 ratio with PKH-labeled platelets at 37°C and 5% CO_2_. After incubation, DCs used for confocal microscopy were washed with PBS + 0.5% BSA and fixed with 3.7% paraformaldehyde (Sigma Aldrich). Subsequently, DCs were quenched with 50 mM NH_4_Cl and labeled for 30 min with unlabeled rabbit antihuman HLA-DR (Abcam, Cambridge, UK) in PBS with 3 mg/ml human gamma globulin. After washing, DCs were incubated 45 min with Alexa 647-labeled anti-rabbit Ig (ThermoFisher, Bleiswijk, The Netherlands) supplemented with 1 µg/ml DAPI nuclear counterstain (Life Technologies). Subsequently cells were washed, taken up in 10 µl Mowiol mounting medium (Calbiochem, EMD Millipore, Billerica, MA, USA) and put on a cover slip after which samples were analyzed using confocal microscopy (LSM 510 META, Zeiss, Breda, The Netherlands). Alternatively, DCs used for image stream analysis were washed with PBS + 0.5% BSA supplemented with 5 mM EDTA (Merck Millipore) to release surface bound platelets after which they were fixed with 3.7% paraformaldehyde (Sigma Aldrich). Subsequently, DCs were incubated at RT with FITC-labeled anti-CD61 (Beckman Coulter) and APC-labeled anti-HLA-DR (both from BD Biosciences, Breda, The Netherlands). After 30 min, cells were washed and analyzed using imaging flow cytometry (ImageStream^®^X Mark II Imaging Flow Cytometer, Merck Millipore) and IDEAS Application (IDEAS software V6.1.303.0, Merck Millipore). Gating strategy is presented in Figure S1 in Supplementary Material, and involved selection of single DCs (aspect ratio intensity vs area; for bright field and APC/BV650 channel), DCs and platelets in focus (gradient RMS; for bright field, APC/BV650 channel and PKH channel), and exclusion of false positive cells (defined as intracellular fluorescence of the platelet-specific anti-CD61 membrane staining).

### Antigen Presentation

The presentation of platelet-derived peptides was performed as previously described (36). In short, 5 × 10^6^ DCs were incubated in a 1:40 ratio with freshly isolated platelets at 37°C and 5% CO_2_. After 5 h of incubation, DCs were matured for 19 h with 1 µg/ml lipopolysaccharide (LPS, Sigma Aldrich) and 1% FCS. Subsequently, the DCs were collected and resuspended in lysis buffer [10 mM Tris (Merck Millipore), 0.25% octyl-b-d-glucopyranoside, 1% sodium deoxycholate and protease/phosphatase inhibitor cocktail (all three from ThermoFisher)]. Lysates were then clarified by centrifugation at 20,000 *g* for 15 min at 4°C, and the supernatants were incubated with 300 µl of Sepharose beads coupled with anti-HLA-DR antibody L243 to immunoprecipitate HLA-DR/peptide complexes. After overnight incubation, samples were washed twice with fresh lysis buffer and five times in 10 mM Tris. Bound HLA-DR/peptides were eluted from the beads using 10% acetic acid (Sigma Aldrich) and desalted using C18 STAGE-Tips (3M Science) before mass spectrometry analysis.

### Mass Spectrometry Data Acquisition

Peptides were separated by nanoscale C18 reverse phase chromatography coupled on line to an LTQ Orbitrap XL mass spectrometer (Thermo Scientific) *via* a nanoelectrospray ion source (Nanospray Flex Ion Source, Thermo Scientific). Peptides were loaded on a 20 cm 75–360 µm inner–outer diameter fused silica emitter (New Objective) packed in-house with ReproSil-Pur C18-AQ, 1.9 µm resin (Dr. Maisch GmbH). The column was installed on a Dionex Ultimate 3000 RSLC nanoSystem (Thermo Scientific) using a MicroTee union formatted for 360 µm outer diameter columns (IDEX) and a liquid junction. The spray voltage was set to 2.15 kV. Buffer A was composed of 0.5% acetic acid in water and buffer B of 0.5% acetic acid, 19.5% water, 80% acetonitrile (Biosolve Valkenswaard, The Netherlands). Peptides were loaded at 300 nl/min at 4% buffer B, equilibrated for 10 min at 4% buffer B (0–10 min), and eluted by increasing buffer B from 4 to 50% (10–50 min), followed by a 3 min wash to 90%, 2 min hold at 90%, a 2 min ramp back to 4%, and a 13 min regeneration at 4%.

Survey scans of peptide precursors from 300 to 2,000 *m*/*z* were performed in the Orbitrap at 30K resolution. Tandem mass spectrometry of the five most intense precursors was performed by isolation with isolation width 1.0, CID fragmentation with normalized collision energy of 35, and rapid scan mass spectrometry analysis in the ion trap. The dynamic exclusion duration was set to 30 s with a 10 ppm tolerance around the selected precursor and its isotopes. Monoisotopic precursor selection was turned on. The MS^2^ ion count target was set to 500, and the max injection time was 30 ms. Only those precursors with charge state 2 and up were sampled for MS^2^. All data were acquired with Xcalibur software. Data files were analyzed with Proteome Discoverer software, version 1.4 (Thermo Scientific) against a human FASTA database (uniProt organism 9606 + keyword 0181) with a 20 ppm tolerance for precursor mass and 10 ppm tolerance for fragment mass. Modifications used were static carbamidomethyl (+57.021 Da) on cysteines and dynamic oxidation (+15.995 Da) on methionine. Only peptides with a high confidence (FDR threshold 0.05%) were considered for protein scoring.

### Human Platelet Antigen 1a (HPA-1a)-Specific T Cell Responses

To investigate if antigen uptake by DCs resulted in immune responses, 5 × 10^3^ HLA-DRB3*01:01 DCs were first incubated 4 h in 1:100 ratio with freshly isolated platelets (untreated, activated and/or apoptotic) in Cellgro DC serum-free medium (Cellgenix) at 37°C and 5% CO_2_. If indicated, DCs were harvested and washed twice at 300 *g* using IMDM culture medium (Lonza, Breda, The Netherlands) supplemented with 5% pooled human serum, 5% fetal calf serum (Bodinco, Alkmaar, The Netherlands), IgG-depleted transferrin, 2-mercaptoethanol (βME), 100 U/ml penicillin, and 100 U/ml streptomycin (both from Gibco/Thermo Fischer Scientific, The Netherlands), and replated in a 96-well flat bottom plate. Next, CD4 T cells specific for HPA-1a presented in HLA-DRB3*01:01 [clone D8T108, for isolation and expansion see Ref. (37)] were added in a T:DC ratio of 10:1 in the presence of 500 ng/ml LPS (Invivogen, San Diego, CA, USA). After overnight incubation, culture supernatant was harvested, and IFN-γ production was determined using ELISA (Sanquin Reagents, Amsterdam, Netherlands).

### Statistical Analysis

Graphical presentation and statistical analyses was performed with GraphPad prism v7.02 (GraphPad Software Inc., La Jolla, CA, USA). A repeated measures one-way ANOVA with Tukey posttesting was used to determine statistical differences induced by pathogen reduction in internalization of platelets by DCs, platelet HLA class I expression, activation and apoptosis. Differences in internalization of stored vs fresh platelets were investigated using a paired *T*-test. Furthermore, statistical differences in internalization of apoptotic vs activated platelets or PS- vs phosphatidylcholine-treated platelets and in all reported CD4 T-cell responses were investigated using a repeated measures one-way ANOVA with Tukey posttesting.

## Results

### Platelet Internalization and Presentation in HLA Class II by DCs

**Figure 1:**
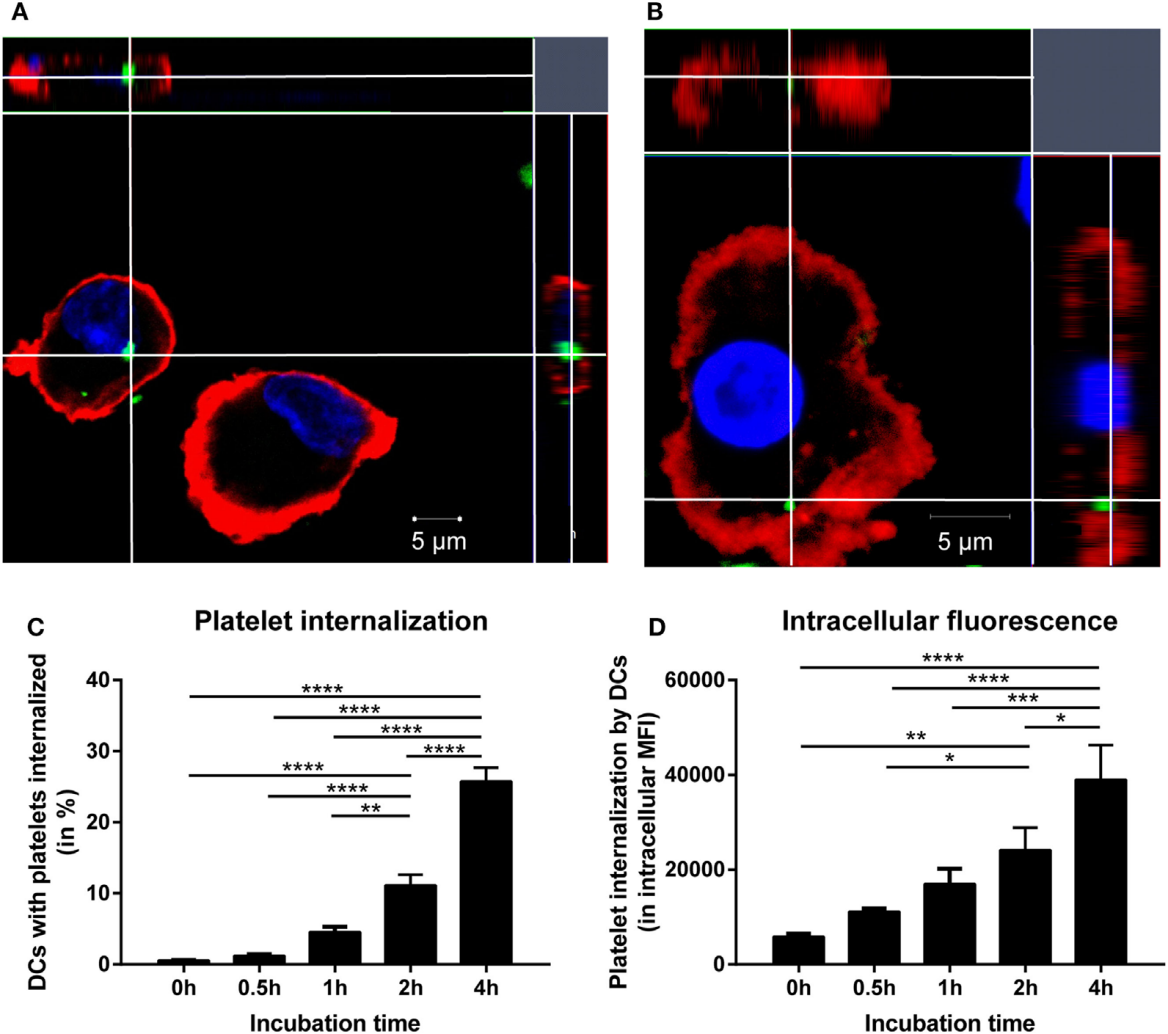
Allogeneic platelets are internalized by dendritic cells (DCs). DCs were incubated 1:40 with PKH-labeled platelets (green). After 4 h incubation, DCs were stained with HLA-DR (red) and DAPI nuclear counterstain (blue) and measured with confocal microscopy (LSM 510 META, Zeiss). To allow visualization from every axis, z-stacks (5–10 slices of 1 μm/slice) were obtained and visualized with an orthogonal presentation **(A,B)**. In addition, using imaging flow cytometry platelet internalization by DCs over time was quantified by determining % of DCs with platelets internalized **(C)** and the intracellular PKH fluorescence in DCs that internalized platelets **(D)** (*n* = 6). One-way ANOVA with Tukey posttesting was used to determine statistical significance (*p < 0.05, **p < 0.01, ***p < 0.001, and ****p < 0.0001).

The first step required for alloimmunization after platelet transfusion is internalization of platelets by antigen-presenting cells. Platelet internalization by DCs was investigated after 4 h incubation using confocal microscopy. z-Stacks were obtained and visualized using orthogonal representation (Figures 1A,B). These images show that DCs are capable of internalizating platelets. Subsequently, platelet internalization by DCs was quantified with imaging flow cytometry and an optimized internalization assay (Figure S1 in Supplementary Material). In time, more DCs internalized platelets (Figure 1C) and each DC internalized more platelets (Figure 1D). In addition, increasing platelet:DC ratio also increased platelet internalization (Figure S2 in Supplementary Material).

Antigen processing and presentation on HLA class II molecules is a second pivotal step to initiate CD4 T cell-mediated alloimmune responses. To investigate if internalized platelets were also processed and presented by DCs in HLA class II molecules, HLA-DR from DCs was immunoprecipitated after incubation with platelets and bound peptides were subsequently analyzed using mass spectrometry. The most abundantly presented peptides were derived from general household proteins, as previously shown (38). To exclude endogenous peptides presented by DCs, all peptides presented by DCs incubated without platelets were removed from the results. After this, 207 and 547 unique presented peptides were identified in two separate experiments, in which we focused on peptides derived from platelet-specific proteins to establish if presentation of platelet-derived peptides occurred. Of note, because HLA class I is expressed by all cells, including DCs, it was not possible to investigate presentation of HLA class I derived peptides. However, peptides from the platelet-specific glycoprotein V were found in each of two independent experiments (Table 1). These peptides were not detected in any of the medium controls, neither by automatic nor by manual inspection of mass spectra. Glycoprotein V was the only platelet-specific protein that was consistently identified, presumably due to limited sensitivity of this assay. The identified glycoprotein V-derived peptides exhibited similar core amino acids between both donors while the flanking regions of these peptides were variable. This is strongly indicative of intracellular antigen processing before their presentation.

**Table 1:** HLA class II presentation of platelet-derived peptides by DCs.

Donor	Glycoprotein V peptides
1	LQELALNQNQLDFLPA
2	VNLQELALNQNQLDFLPA LVNLQELALNQNQLDFLPA LQELALNQNQLDFLPA

### Platelet-Specific T Cell Responses

**Figure 2:**
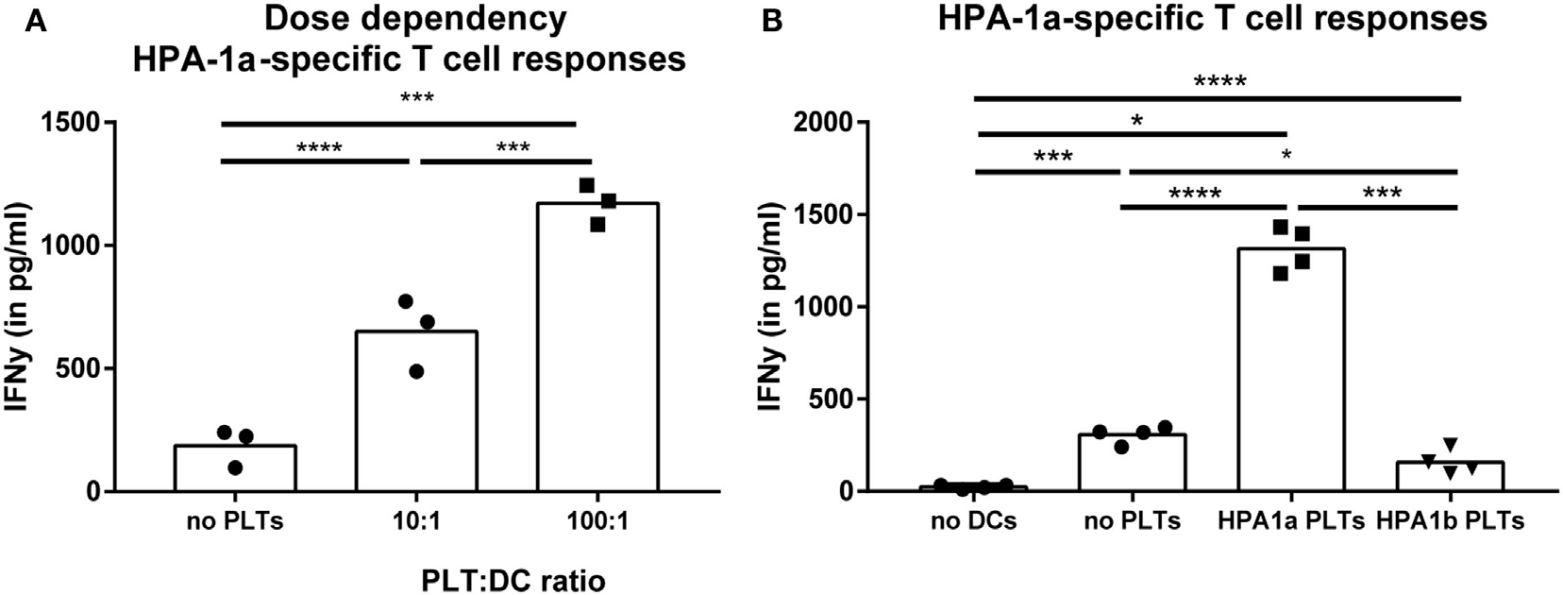
Presentation of platelet-derived peptides by dendritic cells (DCs) induces a CD4 T cell response. Human platelet antigen 1a (HPA-1a)-specific CD4 T cells were incubated with DCs that were pre-incubated 4 h with different PLT:DC ratio with HPA-1a positive platelets [**(A)**, PLT:DC ratio 100:1 and 10:1, *n* = 3] or with either no platelets, HPA-1a negative (i.e., HPA-1b) or positive platelets [**(B)**, PLT:DC ratio 100:1, *n* = 4] after which the HPA-1a-specific T cells were added. After overnight incubation, IFN-γ production by T cells was determined using ELISA. One-way ANOVA with Tukey posttesting was used to determine statistical significance (*p < 0.05, **p < 0.01, ***p < 0.001, and ****p < 0.0001).

Because IgG alloantibody production by B cells requires help from activated CD4 T cells, the potential of DCs to activate platelet-specific CD4 T cells was investigated after incubation of DCs with platelets. To exclude activation of CD4 T cells by antigens derived from a non-platelet source, e.g., contaminating WBCs, T cells recognizing HPA-1a peptides presented in HLA-DRB3 were used. HPA-1a is a minor histocompatibility antigen (22, 23), whose expression in all circulating cells is only reported for platelets (22, 27, 28, 39). Platelet-loaded DCs matched for HLA class II were incubated with the CD4 T cell clone. DCs that were incubated with platelets obtained from HPA-1a positive individuals induced significant IFN-γ production by the HPA-1a-specific CD4 T cells, which increased when more platelets were present (Figure 2A). However, this IFN-γ production was not induced when only platelets were added to the T cells. Although some IFN-γ production was induced when only DCs were added to CD4 T cells, this was similar when DCs were exposed to platelets from HPA-1a negative (thus HPA-1b homozygous) donors (Figure 2B). This demonstrates that DCs internalize platelets and subsequently present platelet-derived peptides in HLA class II to platelet-specific CD4 T cells.

### Product-Related Factors Causing Increased Internalization of Platelets

**Figure 3:**
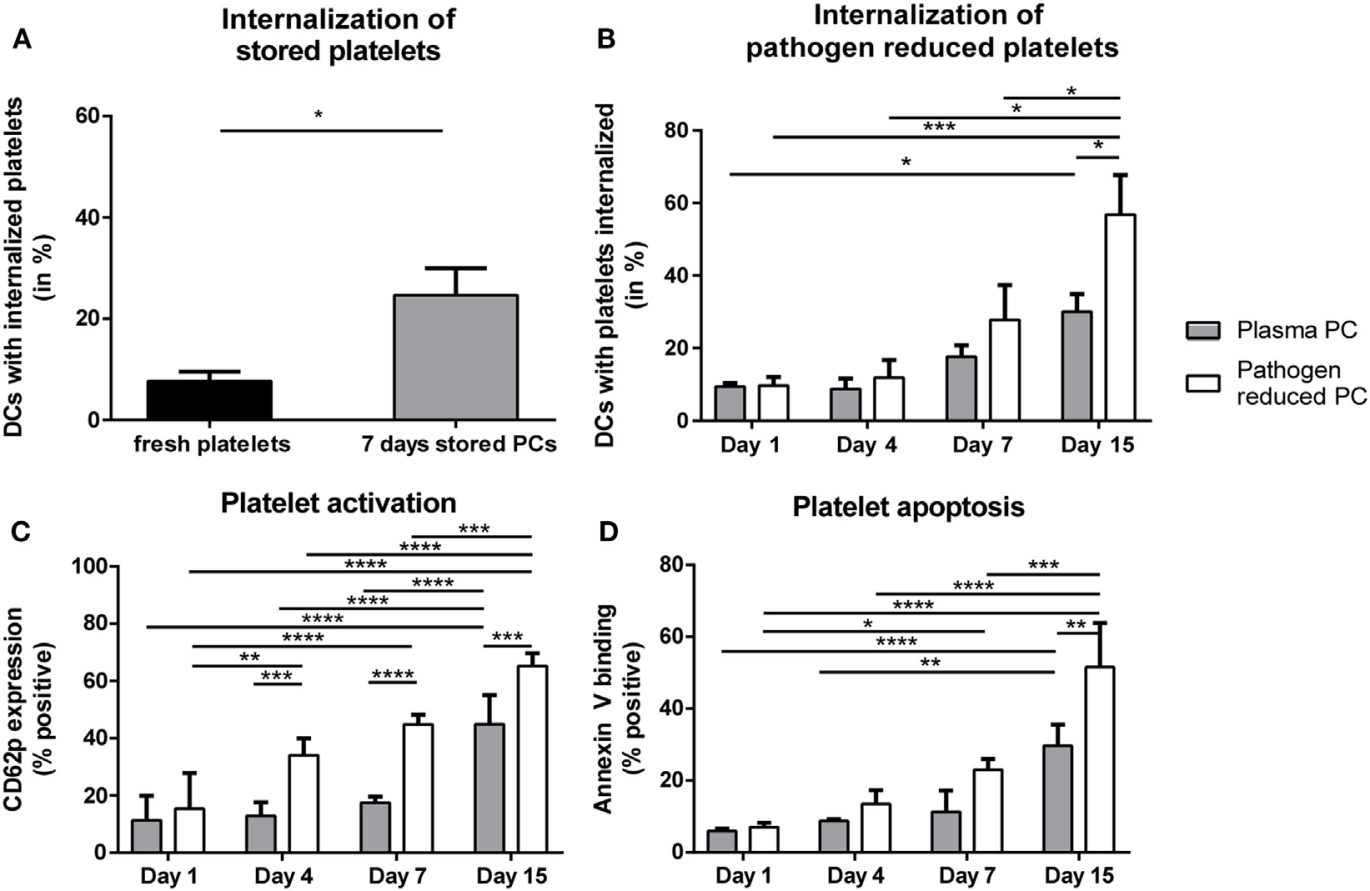
Internalization of allogeneic platelets by dendritic cells (DCs) increases upon storage. Platelets were stored 7 days in platelet concentrates (PCs) under blood bank conditions [**(A)**; *n* = 6]. Alternatively, paired PCs were prepared with or without pathogen reduction and stored for 1, 4, 7, or 15 days [**(B)**; *n* = 4]. Subsequently, stored platelets were incubated 2 h 40:1 with DCs, and internalization of platelets was determined using imaging flow cytometry. In addition, platelet activation [**(C)**, *n* = 3] and apoptosis [**(D)**, *n* = 3] were determined using flow cytometry. Statistical analysis was performed using unpaired *T*-test **(A)** or one-way ANOVA with Tukey posttesting **(B–D)** (*p < 0.05, **p < 0.01, ***p < 0.001, and ****p < 0.0001).

With the evidence above showing the ability of platelet-derived antigens to induce CD4 T cell responses, product-related factors like storage and pathogen reduction could be envisioned as risk factors for platelet dependent alloimmunization, as they may affect platelet internalization and subsequent presentation of platelet-derived antigens in HLA class II. Therefore, internalization of stored platelets with or without pathogen reduction by DCs was quantified. Internalization of platelets stored for 7 days under routine blood bank conditions was significantly enhanced compared with freshly isolated platelets (Figure 3A). Directly after treatment, pathogen reduction by Mirasol did not result in increased internalization of platelets compared with untreated platelets. Upon storage, however, internalization of pathogen-reduced platelets was significantly enhanced compared with similarly stored control PCs (Figure 3B).

### Platelet Apoptosis and Concomitant PS Exposure Enhanced Platelet Internalization

**Figure 4:**
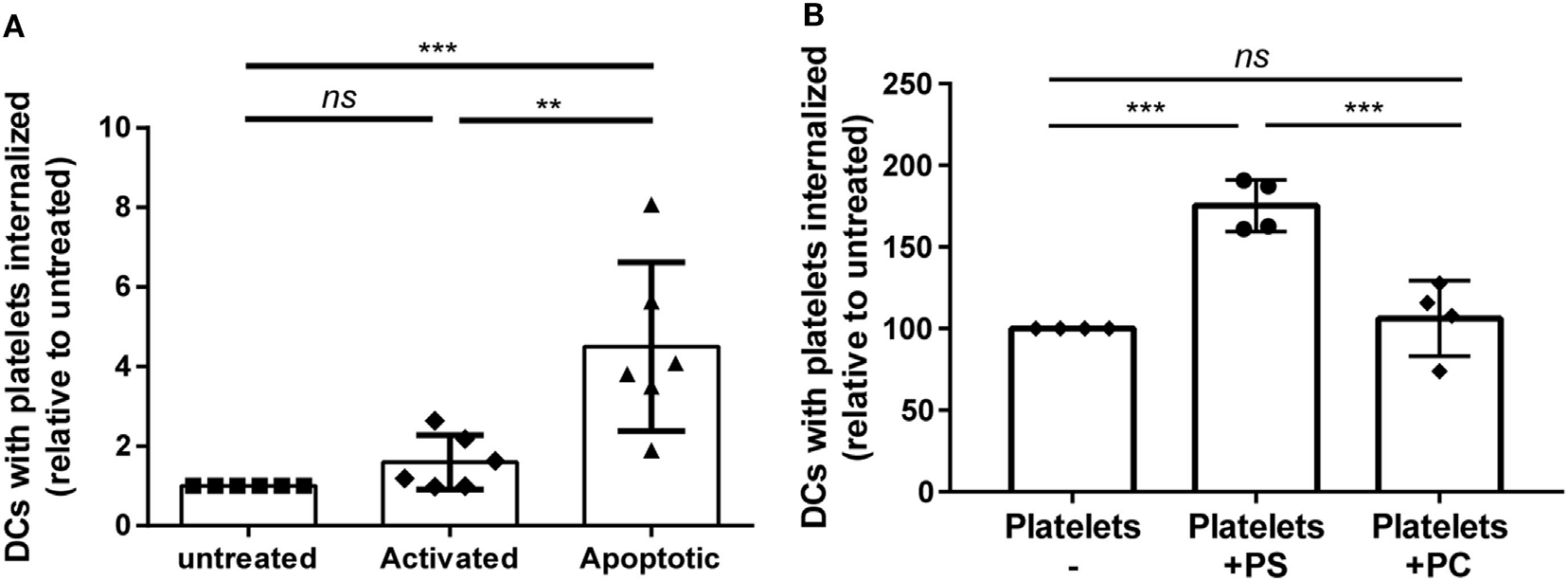
Apoptosis and phosphatidylserine (PS) exposure increase internalization of platelets by dendritic cells (DCs). Freshly isolated platelets were activated, made apoptotic or left untreated [**(A)**, *n* = 6]. Alternatively, platelets were incubated with 250 µg/ml PS or phosphatidylcholine (PC) [**(B)**, *n* = 4]. Subsequently, these platelets were incubated 2 h 40:1 with DCs, and internalization of platelets was determined using imaging flow cytometry. Statistical analysis was performed using one-way ANOVA with Tukey posttesting (**p < 0.01, ***p < 0.001, and ****p < 0.0001; *ns*, not significant).

Storage of platelets resulted in elevated activation and apoptosis (Figures 3C,D). However, HLA class I expression was not significantly affected by storage (Figure S3 in Supplementary Material), as opposed to mouse platelets which completely lose MHC class I expression after 3 days of storage (40). To investigate if platelet activation and/or apoptosis were involved in the increased internalization of stored platelets, freshly isolated platelets were activated or made apoptotic. Activation of platelets resulted in significant CD62P upregulation while annexin V binding was unaffected (Figure S4A in Supplementary Material). Induction of apoptosis in platelets resulted in >90% annexin V positive platelets, while CD62P expression was not affected (Figure S4B in Supplementary Material). Internalization of apoptotic, but not of activated platelets by DCs was increased compared with internalization of untreated platelets (Figure 4A). To investigate the involvement of PS in this enhanced internalization, platelets were incubated with purified PS, which was shown to incorporate into the outer membrane (33). This incubation indeed resulted in incorporation of PS in the outer membrane of platelets, as shown by increased annexin V binding, while increased annexin V binding was not observed after incubation with phosphatidylcholine (Figure S5B in Supplementary Material). Treatment with these phospholipids did not affect platelet activation (Figure S5B in Supplementary Material). Platelets incubated with PS were significantly more internalized compared with phosphatidylcholine incubated or untreated platelets (Figure 4B).

### Platelet Apoptosis Enhanced Platelet-Specific CD4 T Cell Responses

**Figure 5:**
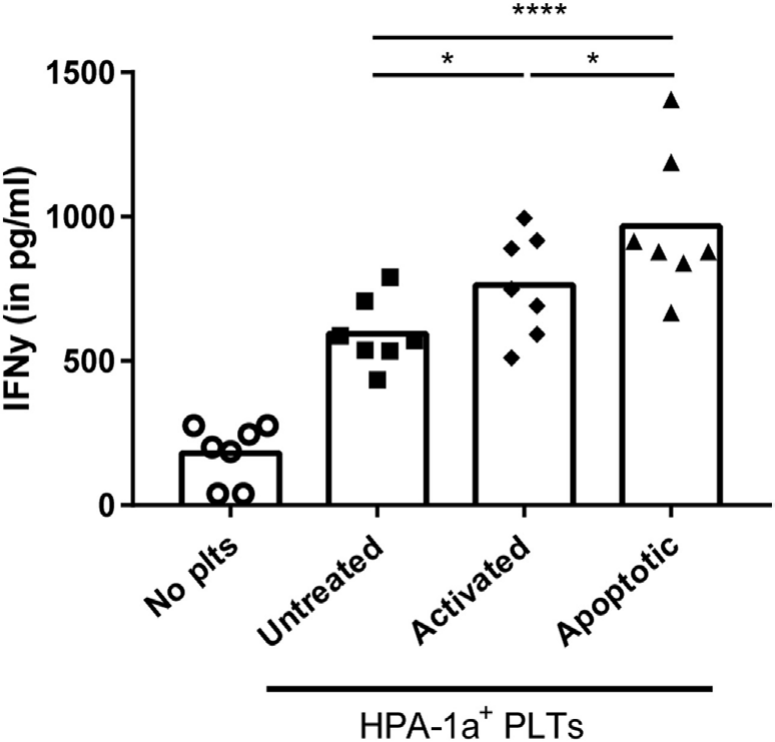
Dendritic cells (DCs) primed with apoptotic platelets induce enhanced CD4 T cell response. DCs were incubated with apoptotic, activated or untreated freshly isolated platelets, washed after 4 h, and subsequently incubated with human platelet antigen 1a (HPA-1a)-specific CD4 T cells. After overnight incubation, IFN-γ by CD4 T cells was determined using ELISA. Statistical significance was determined using one-way ANOVA with Tukey posttesting (*n* = 7; *p < 0.05 and ****p < 0.0001).

Finally, the effect of the increased internalization of apoptotic platelets was investigated on stimulation of CD4 T cell responses using HPA-1a-specific CD4 T cells. While platelet-loaded DCs induced IFN-γ production by platelet-specific (HPA-1a) T cells in all conditions, IFN-γ production was significantly enhanced when DCs were pre-incubated with activated or apoptotic platelets compared with untreated platelets. DCs pre-incubated with apoptotic platelets, however, induced the strongest IFN-γ production by CD4 T cells (Figure 5).

## Discussion

Platelet transfusions can elicit alloimmune responses leading to HLA- or HPA-specific alloantibodies, which result in rapid clearance of opsonized platelets (6). Preventing or minimizing this alloimmunization is not only desirable to prevent refractoriness to platelet transfusions, but also to diminish associated patient morbidity like bleeding complications (1). Currently, it is unknown whether alloimmunization after platelet transfusions is caused by residual WBCs in each blood product or whether platelets themselves contribute to alloimmunization (2, 6, 14, 15, 17–19). This study demonstrates that platelets can be internalized by DCs and presentation of platelet-specific antigen in HLA class II by DCs mediates platelet-specific CD4 T cell activation. Since CD4 T cell help is a prerequisite for differentiation of allo-specific B cells into antibody-producing plasma cells, our data strongly suggest that in addition to WBCs platelets are able to induce alloimmunization, despite contradicting suggestions in literature (17–20). Importantly, our data show that storage-induced apoptosis of platelets increased internalization and platelet-specific CD4 T cell responses and should hence be regarded as a potential risk factor for alloimmunization after platelet transfusions.

Given that platelet concentrates will always contain residual WBCs, in animal or *in vivo* studies it is impossible to dissect the involvement of platelets and WBCs in the induction of the alloimmune response. Therefore, we have chosen an *in vitro* approach to study the direct involvement of platelets in the induction of alloimmunization. The clearest indication for the latter mechanism was derived from the use of a CD4 T cell clone recognizing an HPA-1a peptide. Notwithstanding that HPA-1a is expressed by endothelial cells and trophoblasts, from the circulating cells’ HPA-1a expression is limited to platelets (22, 26–28, 39). As a result, the induced CD4 T cell activation by platelet exposed DCs are due to processing of platelet-derived HPA-1 and subsequent presentation in HLA class II, and thereby a direct indication of a platelet induced alloimmune T cell response. Although HLA class I alloimmunization after platelet transfusions is clinically more relevant than HPA alloimmunization (6, 7), this study used CD4 T cells that respond to HPA-1a antigens to prevent that observed T cell responses are induced by WBCs, which always contaminate isolated platelets. However, platelet-derived antigens are shown to be processed and presented by DCs, suggesting that platelet expressed HLA class I is also processed and presented after platelet internalization. Furthermore, *via* phenomena known as linked recognition, CD4 T cell help can be provided to B cells if the CD4 T cells are specific for the same antigenic complex as the B cell while not necessarily recognizing the same epitope (41). Thus, even if processing and presentation of HLA class I peptides is different as HPA-1a peptides, CD4 T cells specific for HPA-1a peptides may also provide help to HLA-specific B cells.

Internalization, as the first step of platelet-specific alloimmunization, might well be affected by the platelets transfused. Storage of RBCs in this respect has been implicated as a risk factor for alloimmunization (42, 43), and a similar effect could be considered for platelet products. Storage of platelets indeed significantly increased their internalization by DCs as compared with fresh platelets. In addition, Mirasol mediated pathogen reduction, an important new modulation of platelet products (44), further increased the storage-induced internalization, which may be due to enhanced development of storage-induced apoptosis by Mirasol pathogen inactivation (45, 46). Although our study only investigates Mirasol pathogen inactivation, Intercept or Theraflex pathogen inactivation also enhance storage-mediated apoptosis of platelets (44, 46–54) and may therefore also enhance storage-induced internalization of platelets. The potential enhanced risk for alloimmunization after pathogen reduction is contradicted by animal studies that show reduced or absent MHC class I alloimmunization after transfusions with pathogen-reduced platelets (55, 56). This, however, may be explained by the fact that these only use fresh platelets (55, 56), while pathogen reduction did not affect platelet internalization directly after treatment, but rather enhanced the storage-mediated internalization. Interestingly, two clinical trials were recently published that may confirm this hypothesis (57, 58). Namely, one of these studies stored platelets on average for 1 day and showed a protective effect of pathogen inactivation (58, 59), whereas the other trial stored platelets on average for 4 days and did not show any protective effect of pathogen inactivation (57).

As one of the explanations for storage enhanced internalization, we demonstrated that storage-induced PS exposure enhanced internalization and eventual presentation of platelet-derived peptides to CD4 T cells by DCs. Priming of naïve CD4 T cells, however, is not only depending on peptide presentation in HLA class II, but also on co-stimulation and cytokine release by antigen-presenting cells. As a result, the final outcome of peptide presentation to naïve T cells by DCs is likely to be influenced by the overall inflammatory signals present, both by the patients’ inflammatory status and induced by the platelets themselves (60). Corroborating data indeed show the importance of the recipients’ inflammatory status during RBC transfusion wherein pro-inflammatory conditions will skew toward alloimmunization, while an anti-inflammatory environment leads to a more regulatory CD4 T cell response (61, 62). Because PS exposure has been described to aid cell clearance *via* either anti- or pro-inflammatory pathways (63–65), the effect of the increased internalization and presentation of stored platelets on T cell skewing and alloantibody production needs investigation in clinical studies.

As this study provides strong suggestions that storage-induced platelet apoptosis can be a risk factor for alloimmunization after platelet transfusions, studies are warranted that investigate how platelet apoptosis during storage can be abolished or minimized. In addition, conformation by clinical studies is warranted because *in vitro* studies fail to resemble the complexity of *in vivo* immune responses. Finally, the effect of pathogen inactivation on alloimmunization should be investigated preclinical or clinical using routine blood bank conditions, thus with platelet storage that resembles routine practice.

In summary, our study shows that DCs can internalize and present platelet-derived peptides, resulting in platelet-specific CD4 T cell responses. Consequently, factors such as storage of the platelet product, which increase internalization and presentation of these platelets, might increase the risk for alloimmunization, especially when occurring under pro-inflammatory conditions. While confirmation of our findings by clinical studies is warranted, storage-induced apoptosis seems a risk factor for alloimmunization after platelet transfusions.

## Ethics Statement

Studies with samples from volunteer donors being described in this research proposal will be performed according to the Dutch rules and regulations with respect to the use of human materials from volunteer donors. After consultation with the Medical Ethical Committee a system was established for obtaining blood samples for scientific research. This volunteer system is organized according to Dutch regulations and according to the Declaration of Helsinki. This volunteer system certifies, among others, that (1) blood samples used for scientific studies by researchers of the Sanquin Research division were drawn from healthy, anonymized volunteers with written informed consent; (2) no personal characteristics of the volunteers are registered; (3) the volunteers nor those taking the samples know for what project specific samples are used; and (4) allowed annual sample volume and frequency of donation were established after consultation with Sanquin Medical Secretary.

## Author Contributions

AS designed research, performed experiments, analyzed and interpreted data, and wrote the manuscript. IP performed experiments, analyzed data, wrote parts of the manuscript, and reviewed the manuscript. TS isolated and typed CD4 T cells and reviewed the manuscript. PM interpreted data and reviewed the manuscript. SH, JZ, and AB designed research, interpreted data, and critically reviewed the manuscript.

## Conflict of Interest Statement

The authors declare that the research was conducted in the absence of any commercial or financial relationships that could be construed as a potential conflict of interest.
