# Supplementary material for: Storage-Induced Platelet Apoptosis Is a Potential Risk Factor for Alloimmunization Upon Platelet Transfusion
Source: Front Immunol. 2018 Jun 5;9:1251. doi: 10.3389/fimmu.2018.01251 (PMC6008548; doi:10.3389/fimmu.2018.01251)
Supplement: Supplementary file 1 [file image_1.PDF]

Supplementals

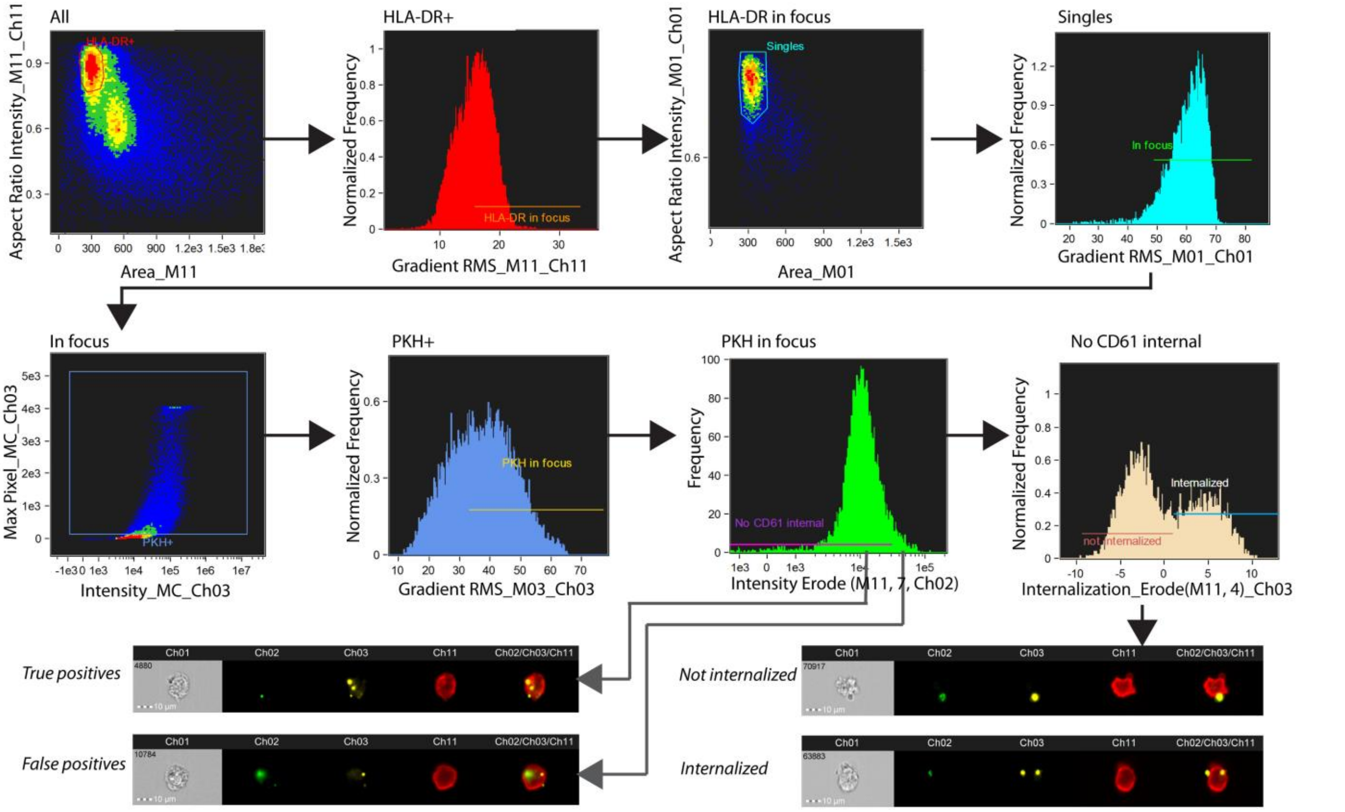

Supplemental figure 1: **imaging flow cytometry gating strategy used to determine platelet internalization by dendritic cells (DCs).** The single cells in focus were first selected using HLA-DR staining and subsequently using bright field. Next, DCs positive for the platelet PKH membrane staining were selected. Subsequently, false positive cells were excluded using CD61 staining. As this CD61 staining was performed after fixation, only surface bound but not internalized platelets can be stained. This allowed for correction of focal plane limitations and seemingly intracellular CD61 events (i.e. false positives) were therefore excluded from further analyses. Typical true and false positive examples are depicted. Finally, internalization of platelets was determined using the internalization feature and the degree of internalization was quantified using intracellular PKH fluorescence.

Parameters used for gating: Area: size of the cell. Aspect Ratio Intensity: circularity of the cell. Gradient RMS: focus of the image. Max pixel: pixel with the highest fluorescence intensity of the cell. Intensity: average fluorescence intensity of all pixels of the cell. Internalization: computes internalization based on intracellular vs extracellular PKH fluorescence intensity.Ch01= bright field, Ch02 = CD61-FITC, Ch03 = PLT PKH membrane staining, Ch11 = HLADR-APC.
