# Supplementary material for: Storage-Induced Platelet Apoptosis Is a Potential Risk Factor for Alloimmunization Upon Platelet Transfusion
Source: Front Immunol. 2018 Jun 5;9:1251. doi: 10.3389/fimmu.2018.01251 (PMC6008548; doi:10.3389/fimmu.2018.01251)
Supplement: Supplementary file 2 [file image_2.PDF]

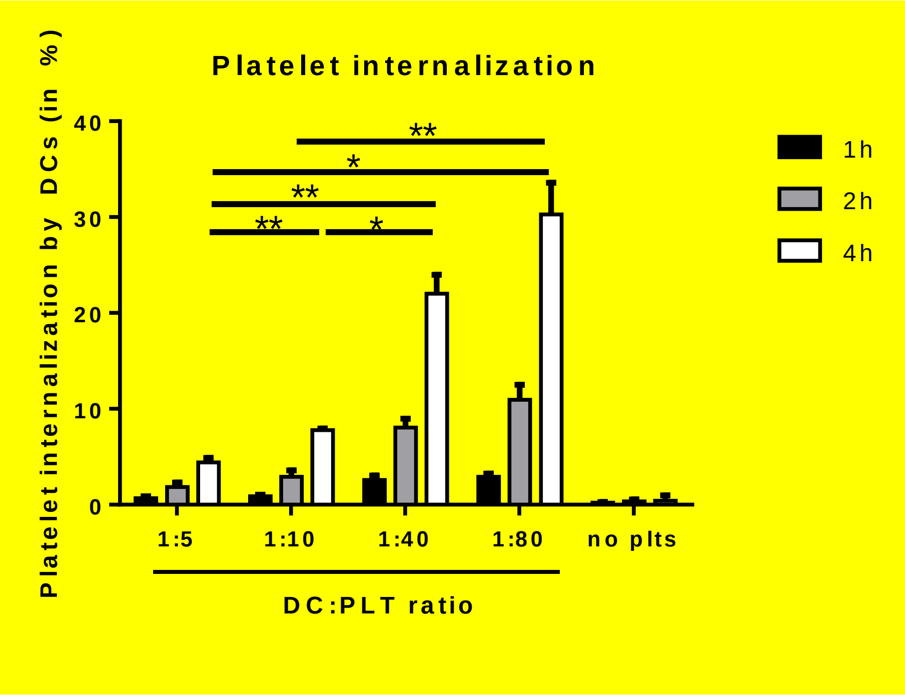

Supplemental figure 2: Platelets internalization by DCs enhances with increasing platelet:DC ratios. Freshly isolated platelets were labelled with PKH and incubated 1-4 hourswith DCs. Subsequently, DCs were harvested, fixed and stained with HLA-DR and CD61. Subsequently, platelet internalization by DCs was quantified over time using imaging flow cytometry. Differences in internalization were determined after 4 hours incubation using one-way ANOVA with Tukey post testing. * $p<0.05$, ** $p<0.01$, *** $p<0.001$, **** $p<0.0001$.
