# Supplementary material for: Storage-Induced Platelet Apoptosis Is a Potential Risk Factor for Alloimmunization Upon Platelet Transfusion
Source: Front Immunol. 2018 Jun 5;9:1251. doi: 10.3389/fimmu.2018.01251 (PMC6008548; doi:10.3389/fimmu.2018.01251)
Supplement: Supplementary file 3 [file image_3.PDF]

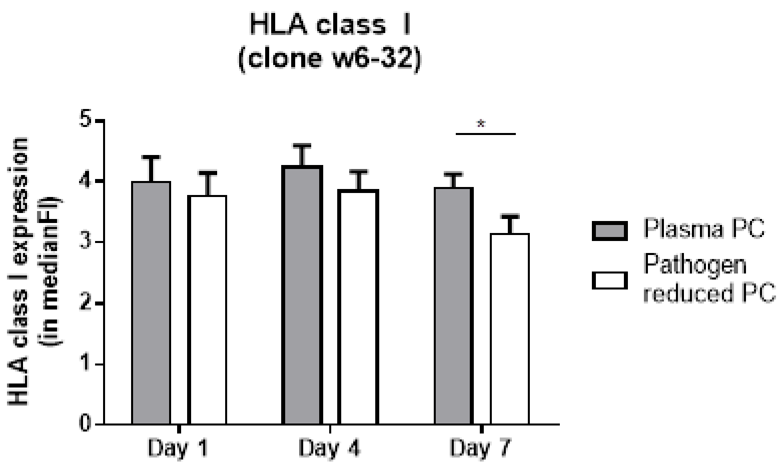

Supplemental figure 3: Effect of storage on expression of HLA class I. Platelets were stored under routine blood bank condition. On day 1, 4 and 7 of storage, HLA class I expression was determined using flow cytometry.
