# Supplementary material for: Storage-Induced Platelet Apoptosis Is a Potential Risk Factor for Alloimmunization Upon Platelet Transfusion
Source: Front Immunol. 2018 Jun 5;9:1251. doi: 10.3389/fimmu.2018.01251 (PMC6008548; doi:10.3389/fimmu.2018.01251)
Supplement: Supplementary file 4 [file image_4.PDF]

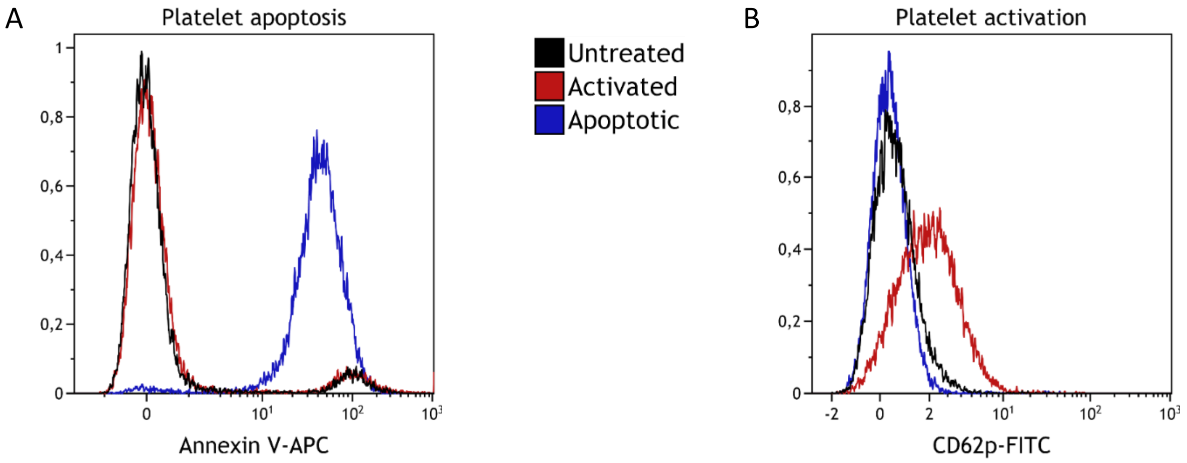

Supplemental figure 4: **Platelet activation and apoptosis after incubation with TRAP-6 or Calimycin.** Freshly isolated platelets were left untreated, activated with TRAP-6 or made apoptotic using Calimycin A23187. After 20 min incubation, annexin v binding (A) and CD62p expression (B) was determined using flow cytometry. Representative graphs from 6 individual experiments are depicted.
