# Supplementary material for: Storage-Induced Platelet Apoptosis Is a Potential Risk Factor for Alloimmunization Upon Platelet Transfusion
Source: Front Immunol. 2018 Jun 5;9:1251. doi: 10.3389/fimmu.2018.01251 (PMC6008548; doi:10.3389/fimmu.2018.01251)
Supplement: Supplementary file 5 [file image_5.PDF]

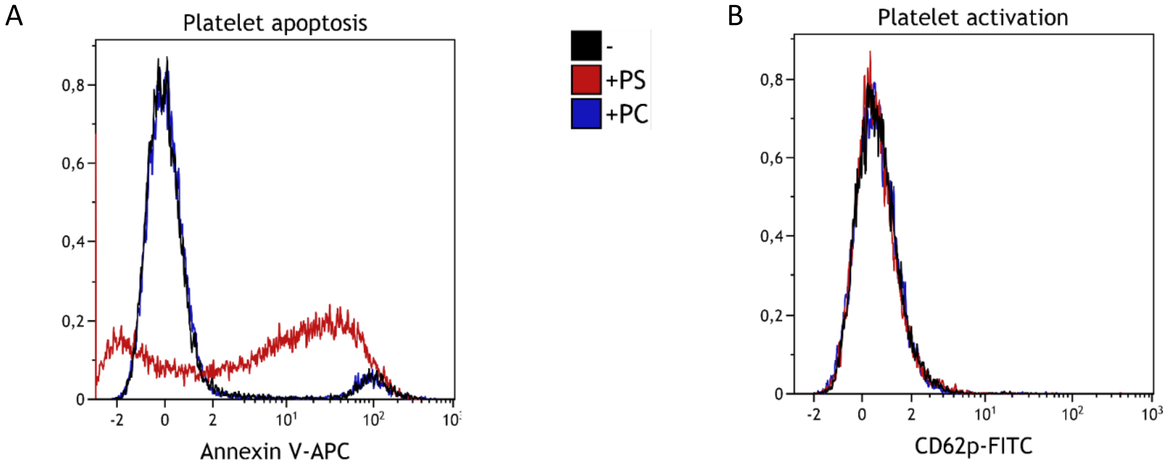

Supplemental figure 5: **Effect of PS and PC on platelet activation and apoptosis.** Freshly isolated platelets were incubated with 250 µg/ml phosphatidylserine (PS) or phosphatidylcholine (PC) or left untreated (-). After 20 min incubation, annexin v binding (A) and CD62p expression (B) was determined using flow cytometry. Representative graphs from 4 individual experiments are depicted.
